# Dynamic Localisation of Mature MicroRNAs in Human Nucleoli is Influenced by Exogenous Genetic Materials

**DOI:** 10.1371/journal.pone.0070869

**Published:** 2013-08-06

**Authors:** Zhou Fang Li, Yi Min Liang, Pui Ngan Lau, Wei Shen, Dai Kui Wang, Wing Tai Cheung, Chun Jason Xue, Lit Man Poon, Yun Wah Lam

**Affiliations:** 1 Departments of Biology and Chemistry, City University of Hong Kong, Hong Kong, China; 2 Centre of Influenza Research and School of Public Health, The University of Hong Kong, Hong Kong, China; 3 School of Biomedical Sciences, The Chinese University of Hong Kong, Hong Kong, China; 4 Departments of Computer Science, City University of Hong Kong, Hong Kong, China; French National Center for Scientific Research - Institut de biologie moléculaire et cellulaire, France

## Abstract

Although microRNAs are commonly known to function as a component of RNA-induced silencing complexes in the cytoplasm, they have been detected in other organelles, notably the nucleus and the nucleolus, of mammalian cells. We have conducted a systematic search for miRNAs in HeLa cell nucleoli, and identified 11 abundant miRNAs with a high level of nucleolar accumulation. Through in situ hybridisation, we have localised these miRNAs, including miR-191 and miR-484, in the nucleolus of a diversity of human and rodent cell lines. The nucleolar association of these miRNAs is resistant to various cellular stresses, but highly sensitive to the presence of exogenous nucleic acids. Introduction of both single- and double-stranded DNA as well as double stranded RNA rapidly induce the redistribution of nucleolar miRNAs to the cytoplasm. A similar change in subcellular distribution is also observed in cells infected with the influenza A virus. The partition of miRNAs between the nucleolus and the cytoplasm is affected by Leptomycin B, suggesting a role of Exportin-1 in the intracellular shuttling of miRNAs. This study reveals a previously unknown aspect of miRNA biology, and suggests a possible link between these small noncoding RNAs and the cellular management of foreign genetic materials.

## Introduction

MicroRNAs (miRNAs) have emerged as ubiquitous regulators of gene expression. The latest version of miRbase reveals 1600 unique human miRNAs (Release 19: August 2012); many of them are highly cell type- or disease-specific [1]. Biogenesis of miRNAs requires multiple enzymatic complexes located in different subcellular compartments. Canonically, miRNAs are transcribed and processed in the nucleus, and matured in the cytoplasm, where they are incorporated into an RNA-induced silencing complex (RISC). Engaged RISCs affect the stability and/or translational potential of mRNA targets [2]–[3]. Bioinformatic estimations suggest that up to one-third of all protein-coding genes in the human genome are under the regulation of at least one miRNA [4].

The biogenesis and processing of miRNAs are precisely controlled, but very little is known about the fate of mature miRNAs beyond their engagement in the RISC. As mammalian miRNAs are extremely stable [5], it is intriguing how cells can adjust the functional pool of specific miRNAs on demand. Parallel analyses of the expression profiles of miRNAs and their target mRNAs tend to show only weak inverse correlations [6]–[7]. In fact, some tissue-specific miRNAs (e.g., miR-363 and miR-124) exhibit no obvious correlation with the expression levels of their target genes. This suggests that the cellular concentrations of individual miRNAs do not necessarily reflect their functional activities. It is probable that not all the mature miRNA molecules are equally active, and there may be a level of “post-maturational” regulation for miRNAs.

The protein complexes responsible for the last steps of miRNA maturation are, at least in cultured cancer cells, located in the cytoplasm. Moreover, miRNAs target RNAs to cytoplasmic foci (P bodies/stress granules) for sequestration and/or degradation [8]–[9]. Since it has been generally established that the cytoplasm is the site of miRNA activity, most researchers assume that all mature miRNAs are located in the cytoplasm. However, direct experimental evidence to support this belief is weak. In fact, as early as 2004, the Tuschl Lab showed that up to 20% of mature mir-21 could be recovered from isolated nuclei [10]–[11]. Hwang et al. (2007) examined the subcellular distribution of 3 mature miRNAs and observed that as much as 32% to 71% of these miRNAs are present in the nucleus [12]. By using high throughput approaches, five recent studies have identified mature human miRNAs in purified nuclei [13]–[16]. The nuclear localisation appears to be restricted to a subset of miRNAs, and an active process that involves CRM1 (expontin 1), but no functional significance has been inferred. More importantly, the Pederson Lab has reported the detection of miR-206 not only in the cell nucleus, but also in the nucleolus of rat myoblasts [17], which suggest a higher level of subnuclear targeting. Their lab has recently extended this finding with a systematic screening of mature miRNAs in rat myoblasts by using a microarray technique [18]. This work reports on the detection of more miRNAs, e.g., miR-351, miR-1 and miR-664 in rat nucleoli.

The accumulation of miRNAs in the nucleolus is a tantalising observation. Research in the past decade has established that the role of the nucleolus, in addition to its well-characterised functions in ribosome biogenesis, is that of a cellular “stress sensor” [19]. Its structural integrity and molecular composition appear to coordinate the phenotypic responses to genotoxic stresses. In this study, we aim to explore the functional connection between miRNA metabolism and nucleolar structure, especially as miRNAs are highly sensitive to environmental stress [9]. The analysis on the dynamics of nucleolar miRNA in cells under stress can provide good insights for uncovering the biological functions of nucleolar miRNAs, and contributing to a more detailed understanding of the cell biology of the nucleolus [20].

We have employed a quantitative real time polymerase chain reaction (qPCR)-based profiling method to screen for mature miRNAs in the nucleolus of HeLa cells. We have identified a group of nucleolar miRNAs that are stably present in the nucleolus of a panel of human cancer cells as well as in primary fibroblasts. To investigate whether nucleolar miRNAs are related to nucleolar stress sensing, we exposed the cells to various reagents, which range from chemicals to nucleic acids, such as DNA oligos, double-strand RNAs, and viral infections. Remarkably, we observed dynamic changes in nucleolar miRNAs, such as miR-484 and miR-191, upon viral infection. To our knowledge, this is the first report that demonstrates a potential linkage between nucleolar miRNAs in combating viruses and mobile genetic elements in maintaining genome integrity.

## Materials and Methods

The protocol for animal work was approved by the Animal Experimentation Ethics Committee of the Chinese University of Hong Kong (Permit Number: 09/050/MIS).

### Antibodies and Oligonucleotides

The antibodies used for Western blotting included: rabbit polyclonal anti-fibrillarin (1∶1000, Santa Cruz), mouse monoclonal anti-α−tubulin (1∶1000, Sigma), mouse monoclonal anti-β-actin (1∶4000, Sigma), and FUS/TLS (1∶1000, Santa Cruz). The antibodies for immunofluorescence co-staining with miRNAs were mouse monoclonal anti-PSP1 (1∶100, gift from Dr. Archa Fox), and mouse monoclonal anti-SC35 (1∶100, Abcam).

The ISH probes used for nucleolar miRNAs were: hsa-miR-191 with a sequence 5′DigN-cag ctg ctt ttg gga ttc cgt tg-3′; hsa-miR-193b with a sequence 5′DigN-agc ggg act ttg agg gcc agt t-3′; hsa-miR-484 with a sequence 5′DigN-atc ggg agg gga ctg agc ctg a-3′; and hsa-miR-574-3p with a sequence 5′DigN-tgt ggg tgt gtg cat gag cgt g-3′. Mature miR-20a was used as the cytoplasmic control which showed no expression in the nucleolus, and its sequence is 5′DigN-cta cct gca cta taa gca ctt ta-3′. The sequences of these miRNAs can be found in the miRbase (http://microrna.sanger.ac.uk). Meanwhile, a customised U3 probe labelled with 5′-fluorescein was used as the nucleolar control, its sequence was 56-FAM/ggt ttt cgg tgc tct aca cgt t-3′, and also with a scrambled negative miRNA control, and its sequence was 5′DigN-g tgt aac acg tct ata cgc cca-3′. The sequence of Actin primers human: Forward primer 5′-cac tct tcc agc ctt cct tcc-3′, Reverse primer 5′-agg tct ttg cgg atg tcc ac-3′. The sequence of GAPDH primers human: Forward primer 5′-gaa ggt gaa ggt cgg agt-3′, Reverse primer 5′-gaa gat ggt gat ggg att tc-3′.

### Cells, Transfection and Cell Treatments

Human HeLa cells, H1299, HUH7, MCF7, A549, RPE, and AG06858 human fibroblast cells were obtained from ATCC, and cultured in Dulbecco’s modified Eagle’s medium (DMEM, Life technologies) supplemented with 10% fetal bovine serum (Gibco), 2 mM Glutamax and 1× Anti-Anti (Gibco) at 37°C with 5% CO_2_. To investigate the miRNA stability to various types of stresses, the HeLa cells were treated with an RNA polymerase I inhibitor (ActD, 5 µg/ml), DRB (25 µg/ml), antibiotics (puromycin, 100 µg/ml), UVC exposure (100 J/m^2^), microtubule poison nocodazole (500 ng/ml), and a DNA damage drug (CPT, 10 µM), respectively. The localisation of miRNAs was monitored before and after these treatments. To induce genetic stresses, dsRNA (control siRNA), pcDNA-GFP DNA, and single-stranded DNA oligos (control inhibitor) were transfected into the HeLa cells in the same condition.

### Primary Fibroblasts Isolation

Fibroblasts were isolated from dermal layer of mouse tails. Briefly, BALB/c mouse (∼25 g) was sacrificed by cervical dislocation. Mouse tail was rinse with 70% ethanol, and the tip of mouse tail was cut into 2-mm small pieces and incubated with 0.25% trypsin/PBS for overnight at 4°C. The skin debris was discarded. Isolated fibroblasts was washed with PBS and placed in a 6 well plate containing DMEM medium supplemented with 10% FBS, 2 mM Glutamax and 1× Anti-Anti (Gibco) and cultured for 4 days. The fibroblast was then subcultured on to coverslip, allow grow for another two days for ISH staining.

### RNA Isolation

HeLa nuclei and nucleoli were isolated as previously described [21]. The total RNA from whole cells, and the nuclei and nucleoli of HeLa were extracted by TRIzol Reagent based on the manufacturer’s instructions (life technologies) and quantified by measuring the absorbance at OD260/280 by “Nanodrop” and further examined by gel electrophoresis.

### miRNA qPCR Profiling

RT-qPCR was performed as previously described [13]. Briefly, 1000 ng of the total RNA from whole cells and nucleoli were reverse transcribed by using Megaplex RT (Pools A and B) respectively, followed by real-time amplification by using low density array (TaqMan) hydrolysis probes in Pools A and B v 2.0–two 384-well plates, according to the manufacturer’s instructions (Applied Biosystems). In this study, we define:

Normalised nucleolus/cell ratio = (miRnucleolus/RNU44nucleolus)/(miRcell/RNU44cell)

Where: miRnucleolus = CT value of a miRNA in isolated nucleolus

miRcell = CT value of this miRNA in whole cell

RNU44nucleolus = CT value of RNU44 in isolated nucleolus

RNU44cell = CT value of RNU44 in whole cell

### AGO2 Immunoprecipitation

RISC was immunoprecipitated by using the miRNA Isolation Kit, Human Ago2 (Wako, Japan) following manufacturer’s instruction as follows. HeLa cells harvested from one 10 cm dish were lysed in 1 ml of Cell Lysis Solution. The lysate was then mixed with 50 µl of Anti Human Ago2 Antibody Beads Solution and the mixture was incubated for 4 hours at 4°C by rotation. After that, the beads were washed with 1 ml of Cell Lysis Solution twice. 50 µl of Elution Solution was then added to beads. After transferring the supernatant to a new tube, 350 µl of ultrapure distilled water (Gibco) and 400 µl of phenol: chloroform: isopropanol (25∶24∶1) was added and the tube was centrifuged at 20,000 g for 10 min at room temperature. The supernatant was transferred to a new tube and 3 µl of ethachinmate, 40 µl of 3 M sodium acetate and 1 ml of 99.5% ethanol was added. The tube was centrifuged at 20,000 g for 15 min at 4°C and the pellet was washed with 1 ml of 70% ethanol. After centrifuging the tube at 20,000 g for 10 mins at 4°C, the pellet was air dried for 10 min. The isolated RNA was dissolved in 20 µl ultrapure distilled water (Gibco). Two-step RT-PCR of miR-484 and miR-20a was performed by using Taqman MicroRNA Assays (life technologies) with special designed primers for both target miRNAs for reverse transcription and RT-PCR respectively.

### qRT-PCR

The reverse transcription for both control genes were performed by using High cDNA Reverse Transcription kit (Applied Biosystems) with random RT primers and the RT-PCR was performed by using KAPA SYBR FAST qPCR Kit Master Mix (2×) Universal kit with ROX Reference Dye High (KAPA BIOSYSTEMS) with designed control gene primers.

### In situ Hybridization (ISH) and Microscopy

ISH was performed by using specific DIG-labeled miRNA LNA probes from Exiqon (Vedbaek, Denmark). Briefly, the cells were first fixed in 4% paraformaldehyde (4°C) for 30 min, followed by 70% ethanol (precooled at −20°C) for at least 16 hrs at 4°C. The cells were permeablised with 0.1 Triton X-100 (dissolved in PBS) for 10 min. The washed cells were then prehybridised with a prehybridisation buffer (4× SSC, 25% formamide, 3× Denhardt’s solution, 2% blocking reagents, 0.25 mg/ml yeast tRNA, 0.25 mg/ml salmon sperm DNA) for 30 min at room temperature, followed by hybridisation at 23°C below the Tm of the LNA probe for 2 hrs as previously described [22]–[23]. The cells were subsequently washed with Washing Buffer I (4× SSC with 0.1% Tween 20), II (2× SSC), and III (1× SSC) at the hybridisation temperature. The cells were blocked with a signal enhancer (Life technologies) for 1 hr at room temperature, and then incubated with a mouse anti-DIG antibody at a dilution of 1∶1000 at 4°C overnight. The cells were washed with PBS three times to remove unbounded mouse anti-DIG antibody. Then, the cells were incubated with a fluorescently labelled secondary antibody (dilution 1∶200). The DNA was stained with Hoechst 33258. The samples were mounted on a fluorescent mounting medium (Dako). The images were taken with a Leica TCS-SPE confocal microscope.

To confirm that the ISH signals were indeed from the specific hybridisation of the probes with the target RNA, the cells were grown on glass coverslips, rinsed once with PBS and once with an ASE buffer (20 mM Tris, pH 7.5, 5 mM MgCl_2_, 0.5 mM EGTA), and permeablised by incubation for 20 min at room temperature with either PBS (mock treatment), RNase (100 µg/ml, Life technologies) or proteinase K (100 µg/ml, Life technologies).

To monitor the localisation of newly processed miR-484, HeLa cells were plated at ∼ 2×10^5^ cells/ml into 24 well-plates and pre-miR484 was delivered into the HeLa cells by electroporation through the “NEON” system with the following conditions: 1005 V, 35 ms width, and 3 pulses(life technologies).

### Western Blotting

The samples were mixed with 2× SDS loading buffer, and denatured at 95°C for 10 min. Proteins were separated on 12% Novex precast gel (Life technologies), and transferred to the nitrocellulose membrane. The membrane was blocked with a blocking buffer (5% (w/v) milk, in PBST buffer) for 1 hr. After a brief rinse, the membrane was incubated with the primary antibody in the blocking buffer for 1 hr. The membrane was washed three times with PBS and incubated with a secondary antibody for 1 hr. After a thorough wash, the membrane was developed with an ECL PlusTM detection kit (GE Healthcare). Gels were imaged by using a Fuji LAS4000 mini chemiluminescent imager.

### Viral Infection

For the viral titration, influenza A virus strain A/WSN/1933 (H1N1) and A/Duck/Hong Kong/Y280/97 (H9N2) were titrated by standard plaque assay. Virus infections were carried out at an MOI of 2 and PBS was used for mock infection. Cells were washed with PBS and then infected with influenza virus at the indicated MOIs for 1 hour at 37°C. Cells were washed with PBS and supplemented with virus culture medium-Eagle’s minimum essential medium (MEM, Life technologies, US) supplemented with 1% penicillin-streptomycin, 1 g/ml TPCK-trypsin.Infected cells were incubated for the indicated time periods at 37°C. All work with the infectious virus was carried out at Biosafety Level 2 (BSL-2).

## Results

### Systematic Analysis of the Nucleolar Distribution of miRNAs in HeLa Cells

**Figure 1:**
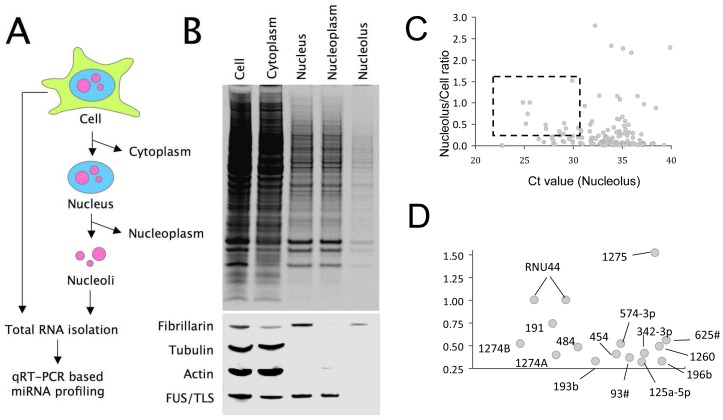
Detection of mature miRNAs in the HeLa nucleolus. (A) Nucleoli of HeLa cells are purified with a two-step protocol. Total RNAs are extracted from the whole cells and purified nucleoli of HeLa, and qRT-PCR based microRNA profiling is performed. (B) The purification and protein recovery of the subcellular fractions are analysed by SDS-PAGE and Western blotting by using antibodies against known subcellular markers. C: Whole cell, Cp: Cytoplasm, Nu: Nucleus, Np: Nucleoplasm, No: Nucleolus. (C) The abundance ratios of the detected miRNAs in nucleolus vs. whole cells are plotted against their abundance in the nucleolus. The box encompasses the miRNAs with high nucleolar abundance (Ct smaller than 30) and nucleolar/cell ratio that exceeds 0.3. (D) Identities of the selected miRNAs.

Since nucleoli can be effectively isolated from human cell lines [21], it is possible to detect the presence of mature miRNAs in these organelles. By using an established protocol, we separated HeLa cells into cytoplasmic and nuclear fractions, which were further separated into nucleoli and nucleoplasm (Figure 1A). Total RNA was then isolated from these fractions. The purity of these subcellular fractions was confirmed by using antibodies against representative marker proteins (Figure 1B), and the integrity of the isolated RNA was validated by gel electrophoresis (Figure S1A). The relative abundance of miRNAs in these fractions was measured by real-time reverse-transcription PCR (qRT-PCR) profiling by using primers specifically designed to amplify the mature form of miRNAs [24]. Among the ∼750 human miRNAs screened, 337 are detectable in the HeLa cells (Table S1). In this study, we have used snoRNA U44 (RNU44), a small RNA known to be highly enriched in the nucleolus as a loading control. In effect, our quantitation approach does not aim to measure the absolute distribution ratio of miRNAs between the nucleolar and non-nucleolar fraction, but to compare the nucleolar localization of miRNAs to that of RNU44. A miRNA with the same level of nucleolar accumulation as RNU44 has a normalized nucleolus/cell ratio of 1. Some miRNAs, such as miR-1275, have nucleolus/cell ratios that exceed one. We interpret these miRNAs as being even more nucleolus-enriched, or more tightly associated with isolated nucleoli than RNU44. This method provides a convenient system of ranking miRNAs according to their nucleolar accumulation. To estimate the contribution of non-nucleolar RNAs in the isolated nucleoli due to contamination during subcellular fractionation, we conducted qRT-PCR with primers specific for two mRNAs (GAPDH and b-actin) on isolated nucleoli and whole HeLa cells. After normalisation with RNU44, the nucleolus/cell ratios of GADPH and b-actin are 0.23 and 0.26 respectively (Table S1). We therefore restricted our further analyses to miRNAs with normalized nucleolus/cell ratios of above 0.30, i.e., those with nucleolar distributions more than 30% of RNU44 and are significantly highly than those of typical mRNAs. Out of the 337 miRNAs detected in HeLa, 48 miRNAs satisfied this criterion. To further eliminate potential false-positives, we restricted our analysis to the miRNAs in which the cycle thresholds (Cts) in nucleolar fractions are below 30 cycles, as the quantitation of nucleolus/cell ratios can be highly inaccurate for non-abundant miRNAs (Figure 1C). Even under such stringent criteria, 11 miRNAs can be identified as miRNAs that are detectable in the nucleolus with very high levels of confidence (Figure 1D, Table S2). Since the detected nucleolar contents of many of these RNAs are very high, we termed these 11 miRNAs as “nucleolar miRNAs”.

Analyses of the sequences, putative targets and chromosome locations of these miRNAs showed no obvious similarities (Table S2). In consistent with Liao et al [14], we failed to observe the 3′ end hexanucleotides, reported by Hwang et al [12] to be important in the nuclear localization of miRNAs, in these nucleolar miRNAs, implying that a mechanism other than that directed by hexanucleotides is involved in the accumulation of miRNAs in the nucleolus. Furthermore, the nucleolar distribution of miRNAs is not significantly correlated with their cellular abundances (Figure S2). Interestingly, two of the identified miRNAs, miR-1274a and miR-1274b, have been recently identified as degradation products of tRNA [25] and are removed from the list. Our result nevertheless suggests potential nucleolar roles of tRNA-derived RNA fragments. Apart from these two RNAs, other nucleolus-localised miRNAs do not share significant homologies with known tRNAs (data not shown).

### Nucleolar miRNAs in Human Cells

**Figure 2:**
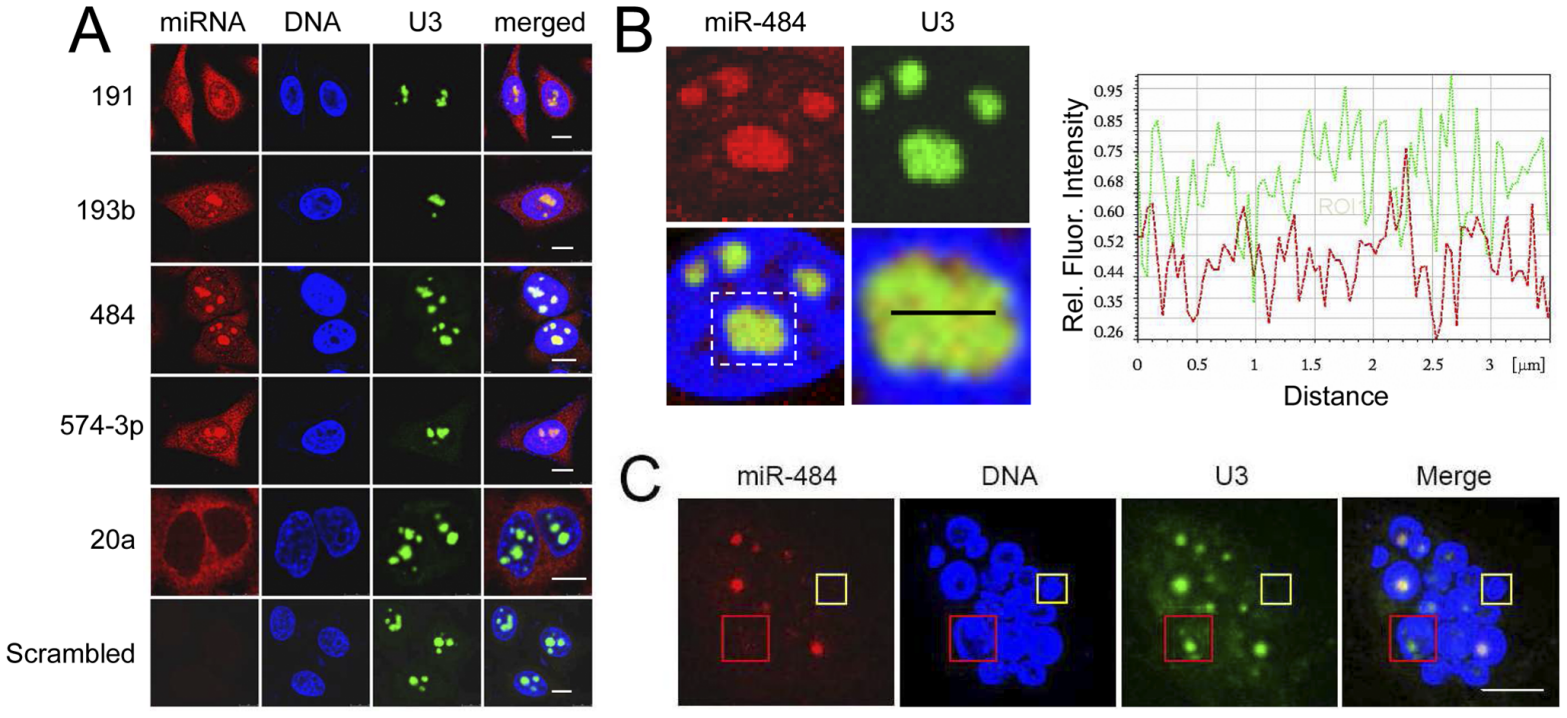
Nucleolar location of mature miR-191, miR-193b, miR-484 and miR-574-3p in HeLa cells. (A) HeLa cells are co-hybridised with DIG-labeled miRNA probes (red) and an FITC labelled probe for U3 snoRNA (green). Four nucleolar microRNAs; miR-191, miR-193, miR-484, miR-574-3p, are examined for their nucleolar location. Cytoplasmic microRNA control miR-20a and a scrambled negative (f) control are also included. The nuclei are stained with Hoechst 33258 (blue). (B) Line profiles of the fluorescence signals of miR-484 (red) and U3 snoRNA (green) in the nucleolus. (C) Micronucleated RPE cells labelled with probes for miR-484 (red) and U3 (green). The micronuclei are stained with Hoechst 33258 (blue). The yellow box highlights a micronucleus without a nucleolus. The red box shows a micronucleus with a nucleolus, but with no miR-484 staining. Scale bars: 10 µm.

Next, we validated our PCR-based observations by using in situ hybridisation, thus avoiding the potential errors intrinsic to subcellular fractionation. The small size of mature miRNAs presents specificity problems for ISH detection. In this study, we used locked-nucleic acid (LNA)-modified oligonucleotide probes, which are superior to conventional probes in their thermal stability, enabling a much higher Tm (as high as 80°C). This minimises non-specific binding for short oligo hybridization. This technology has been used in a large number of studies on the detection of mature miRNAs [26]–[27]. To counter-stain the nucleolus, we performed a dual coloured ISH, in which U3 snoRNA was used as the nucleolar marker. Four nucleolar miRNAs; miR191, miR-484, miR-574-3p and miR-193b, were tested in our ISH experiments, and all four miRNAs exhibit strong nucleolar localisation (Figure 2A). The miRNA identified in the qPCR screen to be nucleolus-excluded, miR-20a, was indeed found by ISH to be mostly cytoplasmic. A control probe, made up of a scrambled sequence, showed no detectable hybridisation (Figure 2A). The ISH signal in the nucleolus was eliminated by RNase treatment, confirming that the nucleolar signal was due to the hybridization of the probes to their RNA targets. Protease treatment did not remove the hybridization signal but led to a dispersal of the staining pattern, possibly as a result of the general degradation of cellular organisation (Figure S3A). Over-expression of the precursor of miR484 in the HeLa cells increased the nucleolar level of miR-484 (Figure S3B), which suggests that the detected nucleolar signals are indeed a result of the maturation of this precursor.

The mammalian nucleolus comprises three major compartments, fibrillar centre (FC), dense fibrillar component (DFC) and granular component (GC) [28]. Nucleolar miRNAs display a intranucleolar pattern similar to that of the muscle-specific miR-206, a nucleolar miRNA colocalised with 28S RNA in the GC region [17]. However, we observed subtle differences in the distribution of the ISH signals from miR191 and that of U3 snoRNA, another known GC component (Figure 2B), which suggest that nucleolar miRNAs and snoRNAs may occupy distinct sub-regions of the GC. We then investigated the location of miR-484 in the micronuclei that did not contain nucleoli. Retinal pigment epithelial (RPE) cells were chronically exposed to a low concentration of the microtubule poison nocodazole in order to generate micronucleated cells. Each micronucleus encompasses a subset of human chromosomes [29]–[30]. The micronucleus with no nucleolus appeared to be devoid of miR-484 (Figure 2C, yellow box), which suggests that the nuclear location of this miRNA may depend on the presence of nucleolar organiser regions (NORs). Intriguingly, in some micronuclei with clearly visible nucleoli, as judged by the presence of U3 snoRNA, there was no detectable miR-484 (Figure 2C, red box). This suggests that the potential involvement of other factors in the nucleolar targeting of miR-484.

**Figure 3:**
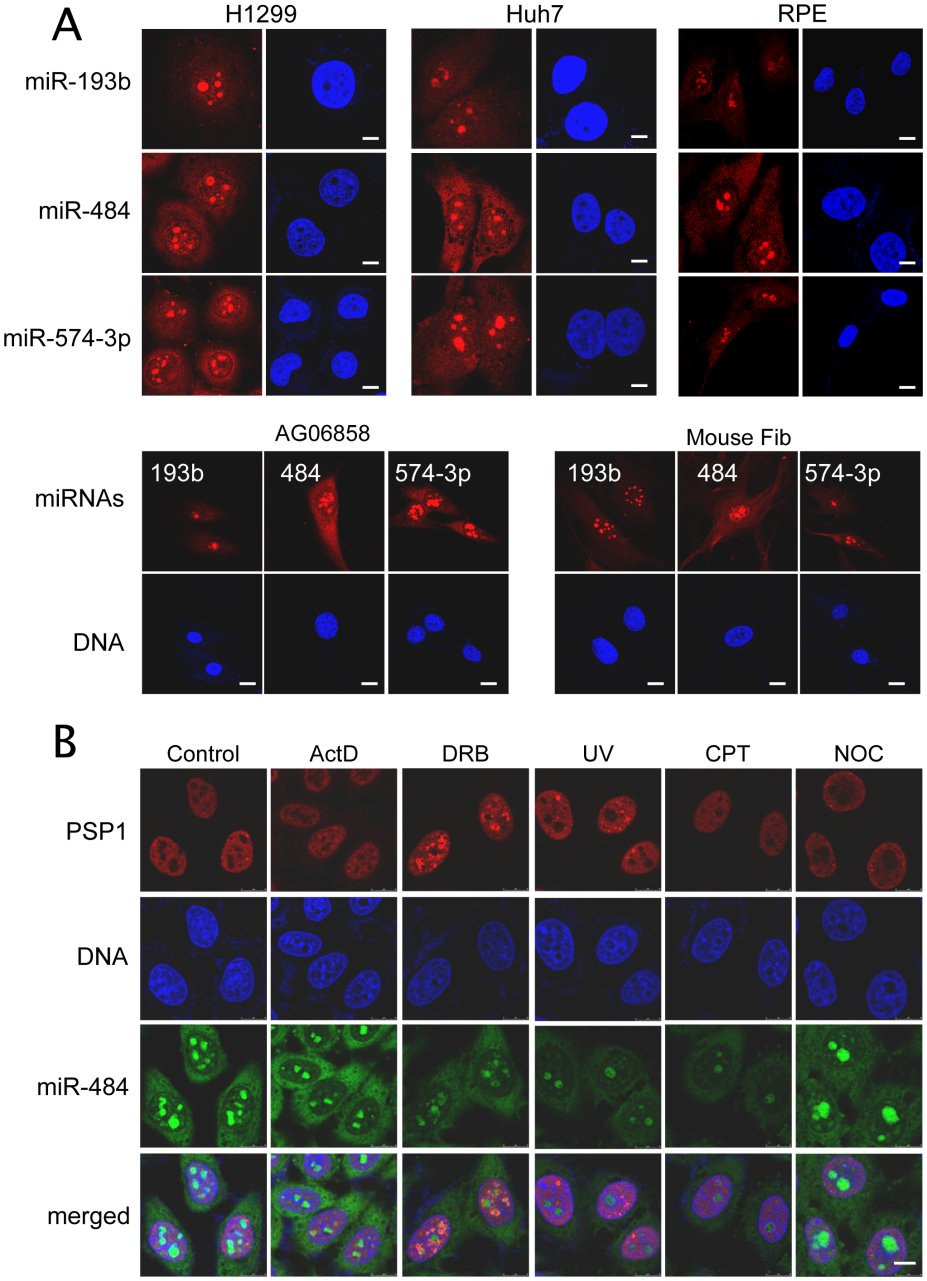
Nucleolar miRNAs are a widespread phenomenon. (A) Localisation of miR-193b, miR-484 and miR-574-3p is examined in lung cancer cells H1299, liver cancer cells Huh7, RPE cells, human adult fibroblast AG06858 and primary mouse adult fibroblast. (B) Intracellular distribution of miR-484 (red) is detected in the HeLa cells 8 hours after exposure to UV (100 J/m^2^), or treated with ActD (5 µg/ml, 2 hrs), or DRB (25 µg/ml, 2 hrs). PSP1 (green) is used to confirm the effect of the treatments. The cell nuclei are counterstained with Hoechst 33258 (blue). Scale bars: 10 µm.

We examined the subcellular distribution of these miRNAs in other human cell lines (Figure 3A), such as lung cancer cell line H1299 and liver cancer cell line Huh7 and in non-transformed cells including RPE cells, human fibroblast cell AG06858 and in freshly isolated mouse primary fibroblasts. The nucleolar location of these miRNAs was found in all the cell lines tested and homogeneously detected in all the cells in the culture, which suggest that the distribution of these miRNAs is independent of cell cycle stages or other intra-population variations. In addition, we showed the presence of these miRNAs in the nucleolus of the canine cell line MDCK (Figures S5B–C), which suggests a level of evolutionary conservation of the nucleolar localisation of these miRNAs. Taken together, the nucleolar accumulation of miRNAs appears to be a widespread phenomenon in cultured mammalian cells.

### Nucleolar miRNAs are Less Engaged in RISC

**Figure 4:**
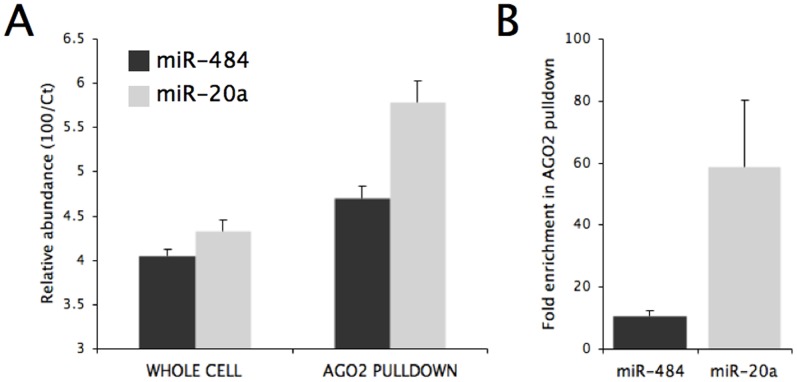
miR-484 and miR-20a bind to RISC with different efficiency. (A) Relative abundance of miR-484 and miR-20a determined by qRT-PCR in unfractionated HeLa cells and in AGO2 immunoprecipitates. (B) Enrichment factors of miR-484 and miR20a in AGO2 immunoprecipitates, converted from the qRT-PCR data.

The differential subcellular locations of miRNAs suggest that different miRNAs may adopt different post-maturation fates. The most well-characterised function of miRNAs is their RNA suppression activities through the incorporation into RISC. We asked whether nucleolar miRNAs are engaged in RISC to the same extent as miRNAs that are predominately cytoplasmic. As shown in Figure 4A, the total cellular content of miR-484 and miR-20a, miRNAs that are shown to be clearly nucleolar and cytoplasmic respectively (Figure 2A), are comparable. However, significantly more miR-20a was detected in RISC isolated by AGO2 immunoprecipitation than miR-484. When these qRT-PCR data were translated into fold-enrichment of the respective miRNAs in the isolated RISC (Figure 4B), we observed that miR-20a was about 6 times more likely than miR-484 to be co-purified with RISC. This result suggests that miR-484, and possibly other nucleolar miRNAs, are less efficient than cytoplasmic miRNAs in their incorporation in RISC, possibly because of the inaccessibility of the protein components of RISC which are predominately cytoplasmic.

### Nucleolar miRNAs are Stably Associated with the Nucleolus

Apart from its well-established role in ribosomal biogenesis, the nucleolus is increasingly recognised as the sequestration site for proteins. The reduced efficiency of nucleolar miRNAs in binding to RISC implies that the nucleolar accumulation may represent a way of sequestrating these mature miRNAs away from their sites of action. To test this hypothesis, we exposed HeLa cells to a variety of chemical stresses known to induce nucleolar disruption [21], [31]–[33], and examined their impact on the location of one of the nucleolar miRNAs, miR-484. The cells were counter-stained with an antibody against PSPC1, the marker protein of paraspeckles, a structure highly sensitive to cellular stress [34]. The nucleolar location of miR-484 was not affected by treatments with transcription inhibitors Actinomycin D (ActD), 6-dichloro-1-beta-D-ribofuranosyl -benzimidazole (DRB), UV, Nocadozole and topoisomerase inhibitor Camptothecin (CPT) (Figure 3B). Hence, the accumulation of miR-484, and possibly other nucleolar miRNAs, in the nucleolus appears to be highly resistant to various forms of metabolic perturbations, which implies that these miRNAs are tightly integrated into the nucleolar structure. The stability of the nucleolar localisation of these miRNAs is also illustrated by the fact that their discovery was based on their presence in isolated nucleoli. Their associations with the nucleolar structure must therefore be strong enough to withstand the procedure of subcellular fractionation.

### Nucleolar Location of miRNAs Responds to Exogenous Genetic Materials

**Figure 5:**
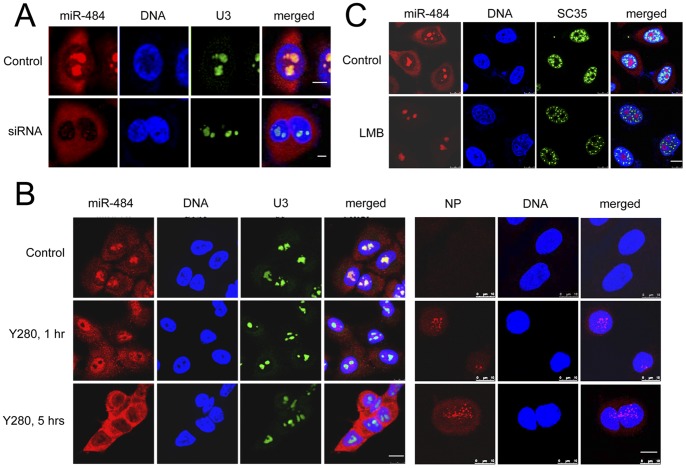
Dynamic nucleolar localisation of miR-484. (A) ISH of miR-484 and U3 in HeLa cells 72 hrs after transfection with a scrambled siRNA. (B) Lung cancer cells A549 are infected with influenza A/Duck/HongKong/Y280/97 (H9N2) strain (MOI = 2) for 1 hr or 5 hrs, respectively. The successful infection of influenza A virus is indicated by viral NP staining. (C) LMB decreases cytoplasm miR-484. The HeLa cells are incubated with or without LMB (20 nM) for 6 hrs, and then fixed for ISH staining. SC35 is a nuclear speckle marker used to confirm the effect of LMB. Cell nuclei are stained with Hoechst 33258 (Blue). Scale bars: 10 µm.

During our experiments on cellular stress, we examined the intracellular distribution of miR-484 in cells challenged with foreign nucleic acids. Unexpectedly, the introduction of a short double stranded RNA (dsRNA) with no known human homology effectively changes the intranuclear distribution of miR-484 (Figure 5A), from nucleolar to cytoplasmic. The change was detectable in a small portion of cells as early as 48 hrs after transfection and pertained for up to 3 days, with no observable changes in cell viability and growth rate (data not shown). This effect on miR-484 localisation was not observed in mock-transfection (Figure S4A, upper panels), and did not depend on the method of transfection, as dsRNA delivered by both electroporation- or Lipofectamine-mediated transfection caused the same changes (data not shown). Similar changes were also observed when cells were transfected with other types of nucleic acid, such as single stranded and plasmid DNAs (Figure S4A–B). In particular, the use of a DNA plasmid that encodes for Green Fluorescent Protein (GFP) allowed us to assess the impact of the amount and the expression of exogenous DNA on the subcellular localization of nucleolar miRNAs. Some of the transfected cells expressed a much higher level of GFP (strong whole cell fluorescence) than others (weaker and predominantly cytoplasmic fluorescence). However, there is no obvious correlation of the level of miR-484 redistribution and GFP expression level (Figure S4A, lower panels). It is possible that the distribution of other nucleolar miRNAs also respond in the same manner, as another nucleolar miRNA, miR-191, also re-distributed following transfection (Figure S4B).

Our data demonstrated the dramatic impact of exogenous genetic materials on the intracellular distribution of mature miRNAs. However, transfection is a non-physiological process, in which the level of cellular stress and the amount of nucleic acid delivery are difficult to control. To confirm the effect of exogenous nucleic acids on miRNA localisation, we adopted a more physiological approach, which involves the use of viral infection. We infected A549, a lung cancer cell with A/Duck/Hong Kong/Y280/97 (H9N2) influenza virus and then examined the localisation of miR-484. As shown in Figure 5B, miR-484 shows a dramatic redistribution from the nucleolus to nucleoplasm, and then to the cytoplasm within 5 hrs after infection. Viral infection of the cells was confirmed by nucleoprotein (NP) staining (Figure 5B). Similarly, the translocation of miR-484 was also observed after infection with a human influenza virus (A/WSN/33), in the A549 cells (Figure S5A) and in cell lines, MDCK (Figures S5B–C) and HeLa (data not shown). These data suggest that the nucleolar distribution of miRNAs, although highly resistant to a wide range of chemically induced stresses, is sensitive to the specific genomic stress induced by exogenous nucleic acids.

### Subcellular Distribution of Nucleolar miRNAs Depends on Exportin-1 Activity

The miRNA redistribution after transfection and viral infection suggests a dynamic distribution of these miRNAs between the nucleus and cytoplasm. Since Exportin-1 can be coprecipitated with AGO-1 and AGO-2 [35], we asked whether this exportin is involved in the regulation of the nucleocytoplasmic partitioning of nucleolar miRNAs. As exportin-1 dependent nuclear export is sensitive to Leptomycin B (LMB) [36], we treated HeLa cells with 20 nM LMB for 6 hrs and then probed for the location of miR-484 by ISH. As shown in Figure 5C, there is virtually no cytoplasmic miR-484 after LMB treatment, which suggests that this miRNA may shuttle between the nucleus and cytoplasm, mediated by the exportin-1 pathway. Our data corroborate with previous observations in that the nucleocytoplasmic distribution of miR-16 and miR-29b can be changed by LMB [35].

**Figure 6:**
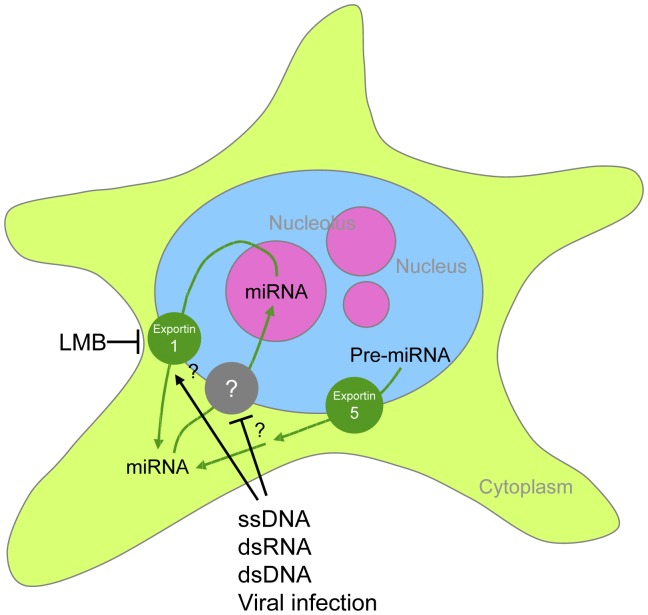
The intracellular dynamics of miRNAs.

Previous studies have established that pre-miRNAs are exported into the cytoplasm via Exportin-5 [37], where they are further processed into mature miRNAs. Our data suggest that a subset of mature miRNAs can be imported to the nucleolus by an unknown mechanism. The nucleolar pool of miRNAs can be re-exported to the cytoplasm via Exportin-1 (Figure 6). The presence of exogenous nucleic acids can effectively disrupt this shuttling process, and deplete the nucleolus of miRNAs. It is possible that these foreign nucleic acids may saturate the nuclear import mechanism of small RNAs, or stimulate the export of miRNAs from the nucleus (Figure 6).

## Discussion

In this study, 11 abundant miRNAs are identified in the nucleolus of HeLa. It is the first work on human cells showing the nucleolar localization of miRNAs. The presence of miRNAs in the nucleolus has been reported in other mammals [17]–[18]. The Pederson Lab identified more than 50 miRNAs that are highly enriched in the nucleolus of rat myoblast cells [18]. Three out of the 11 nucleolar miRNAs identified in our study, including miR-191 and miR-484, are also on their list (Table S1). This is a remarkable level of overlapping, given the cell type-specificity of mammalian miRNAs [38]. We have demonstrated here the nucleolar localisation of mature miRNAs in a wide diversity of human, mouse and canine cell lines, regardless of cell types and transformation status. This suggests that the localisation of miRNAs in nucleoli is a general phenomenon in mammalian cells, rather than a unique property of myogenic cells. The nucleolus is a very dynamic structure [21], [39]. Activities of a wide range of proteins, including MDM2 [40], ADAR2 [41], HAND1 [42] and RelA [43], are controlled by their nucleolar localisation, possibly through a mechanism mediated by a noncoding RNA encoded in the ribosomal intergenic region [44]. Moreover, the nucleolar structure can be reversibly disrupted in response to cellular stress, which allows sequestered nucleolar factors to disperse out [34], [45]–[46]. In this report, we show for the first time that the nucleolar location of mature miRNAs is also highly dynamic. Although nucleolar miRNAs were not affected by metabolic perturbations known to disrupt the nucleolar structure, a single dose of exogenous nucleic acid was sufficient to disperse these miRNAs into the nucleoplasm and cytoplasm. Future studies will include a detailed investigation on the possible roles of the type and sequence of nucleic acids in this phenomenon. The functional significance of this redistribution is still not clear. The need to manage foreign genomes and their degradation products may saturate the same molecular machineries used for the subcellular partitioning of miRNAs. Interestingly, we showed that miR-484, a nucleolar miRNA, is much less efficient in the binding to RISC when compared to miR-20a, a cytoplasmic miRNA. This suggests that the displacement of miRNAs from the nucleolus to the cytoplasm makes them accessible to RISCs and the sites of translational suppression.

The rapid redistribution of miRNAs after influenza A viral infection offers a tantalising perspective on the functional link between the intracellular targeting of miRNAs and the defense against viral infection. Interestingly, IFNAR1, an interferon receptor, is identified by six different miRNA target prediction algorithms as a target of miR-484 (http://www.umm.uniheidelberg.de/apps/zmf/mirwalk/micrornapredicted-target.html). IFNAR1 is known to be down-regulated after influenza A infection [47]–[48], possibly through the activation of miR-484. In future studies, we will experimentally test whether the expression of IFNAR1 is under the control of miR-484. We will over-express or knock down miR-484, and then monitor the potential changes of IFNAR1 level. We will also examine the progression of influenza A infection after the knockdown of nucleolar miRNAs. Investigating the interactions between RNA viruses and the nucleolus will facilitate the design of novel anti-viral therapies, such as recombinant vaccines and therapeutic molecular interventions, and also contribute to a more detailed understanding of the cell biology of the nucleolus [20].

Numerous reports on the profiling of miRNA expression in different tissues have been published. In those experiments, the abundance of a certain miRNA is usually used to correlate with disease states or physiological conditions. However, this popular approach is based on a hidden assumption that the detectable cellular abundance of a miRNA represents its entire functionally active pool. Our data show this is not necessarily the case. Here, we have shown here that miRNAs are dynamically partitioned into different intracellular pools. As the functional differences of miRNAs located in different organelles are unknown, some of these miRNA expression studies may yield potentially misleading conclusions. We believe the subcellular distribution of mature miRNAs shown in this and other reports [13]–[16], [18] should initiate cautions in designing and interpreting miRNA profiling experiments. Notably, miR-191 and miR-484, two of the nucleolar miRNAs revealed in this study, have been suggested as internal references for miRNA profiling [49]–[51], possibly because of their high abundance. Their predominant nucleolar localisation implies these miRNAs may play non-canonical roles and are not suitable as a general reference for the quantitation and characterisation of other miRNAs.

## Supporting Information

Figure S1 Quality of RNA used in the qRT-PCR miRNA profiling experiment. Total RNAs are extracted from whole cells, and purified nuclei and nucleoli and the quality of RNA from the three samples are checked by electrophoresis in agarose gel. “C”, whole HeLa cell, “Nu”, purified HeLa nuclei, “No” purified HeLa nucleoli.
(TIF)

Figure S2 Correlation of the normalized nucleolus/cell ratios of the miRNAs with their Ct values detected in total HeLa cells.
(TIF)

Figure S3 ISH signal of miR-484 is sensitive to RNase A and delivery of pre-miR-484 increases nucleolar miR-484 in HeLa cells. Cells are incubated for 20 min at room temperature with either PBS (mock treatment), RNase A (100 µg/ml, Invitrogen) or Proteinase K (100 µg/ml, Promega) and further fixed for ISH staining. The HeLa cells are also transfected with 25 nM pre-miR-484 by a NEON transfection system. Post transfection (6 hrs), they are fixed and analysed by ISH staining. Nucleolar marker, U3 is in green and cell nuclei are stained with HOECHST33258. Scale bars, 10 µm.
(TIF)

Figure S4 Nucleolars miR-484 and miR-191 are sensitive to exogenous nucleic acids. The HeLa cells were mock-transfected (A, upper panels) or transfected with 1 µg/ml pCDNA-GFP plasmid (A, lower panels) for 72 hrs, or transfected with a single stranded short DNA oligo for 20 hrs, 48 hrs and 72 hrs (B), and fixed and processed for ISH with an miR-484 or miR-191 probe. The expression of the GFP is also captured (A). The cell nuclei are stained with HOECHST33258. Scale bars: A, 25 µm, B, 10 µm.
(TIF)

Figure S5 Nucleolars miR-484 and miR-191 rapidly respond to influenza A virus infection. Influenza A model cell lines A549 - a human lung cancer cell (A) and MDCK- a dog kidney cells (B, C) are infected with influenza A/WSN for different times, fixed and processed for ISH analysis with miR-191 and miR-484 probes. The cell nucleoli are indicated with U3 snoRNA (green) and the cell nuclei are stained with HOECHST33258 (Blue). Scale bars, 10 µm.
(TIF)

Table S1 MiRNAs in HeLa. The expression of 750 human miRNAs in whole HeLa cell and isolated nucleoli were screened. Data was normalized to a nucleolar control snoRNA U44 (RNU44).
(XLS)

Table S2 Nucleolar miRNAs in HeLa. The genomic location, validated targets and sequence of the most abundant 11 nucleolar miRNAs were summarized.
(DOC)
