# Supplementary figures and images for: Dynamic Localisation of Mature MicroRNAs in Human Nucleoli is Influenced by Exogenous Genetic Materials
Source: PLoS One. 2013 Aug 6;8(8):e70869. doi: 10.1371/journal.pone.0070869 (PMC3735495; doi:10.1371/journal.pone.0070869)

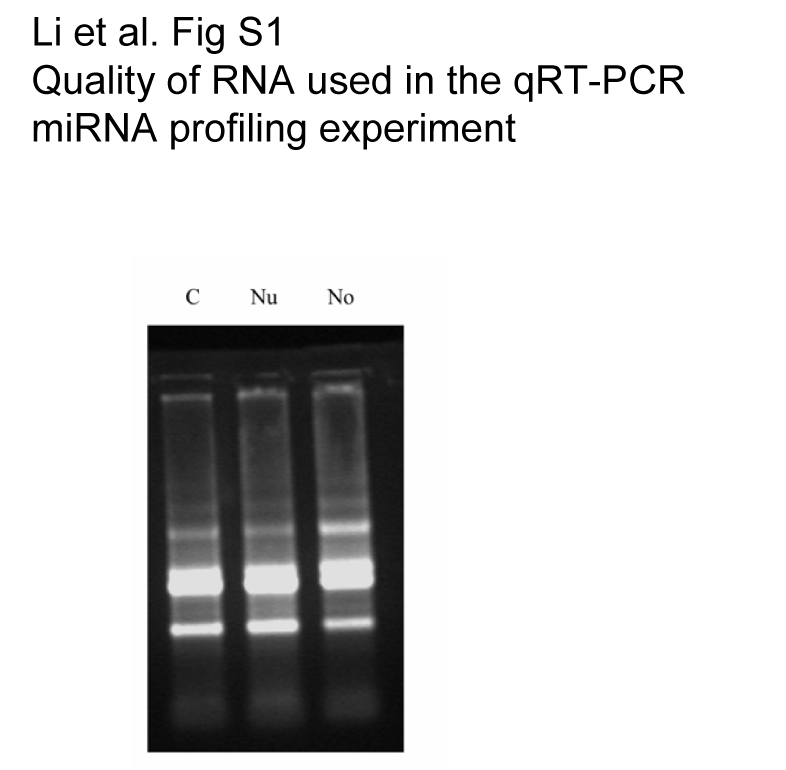

Supplement: Figure S1 — Quality of RNA used in the qRT-PCR miRNA profiling experiment. Total RNAs are extracted from whole cells, and purified nuclei and nucleoli and the quality of RNA from the three samples are checked by electrophoresis in agarose gel. “C”, whole HeLa cell, “Nu”, purified HeLa nuclei, “No” purified HeLa nucleoli. (TIF) [file pone.0070869.s001.tif]

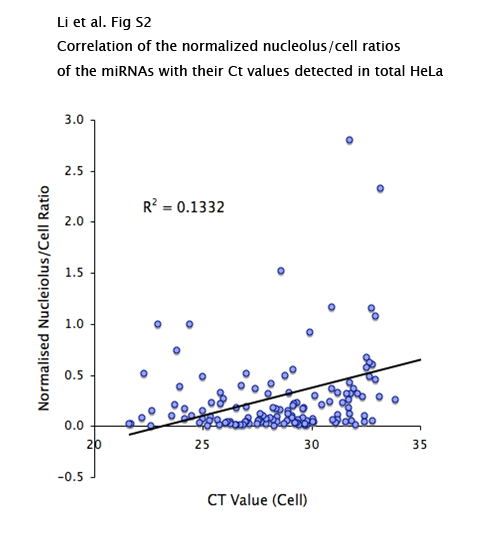

Supplement: Figure S2 — Correlation of the normalized nucleolus/cell ratios of the miRNAs with their Ct values detected in total HeLa cells. (TIF) [file pone.0070869.s002.tif]

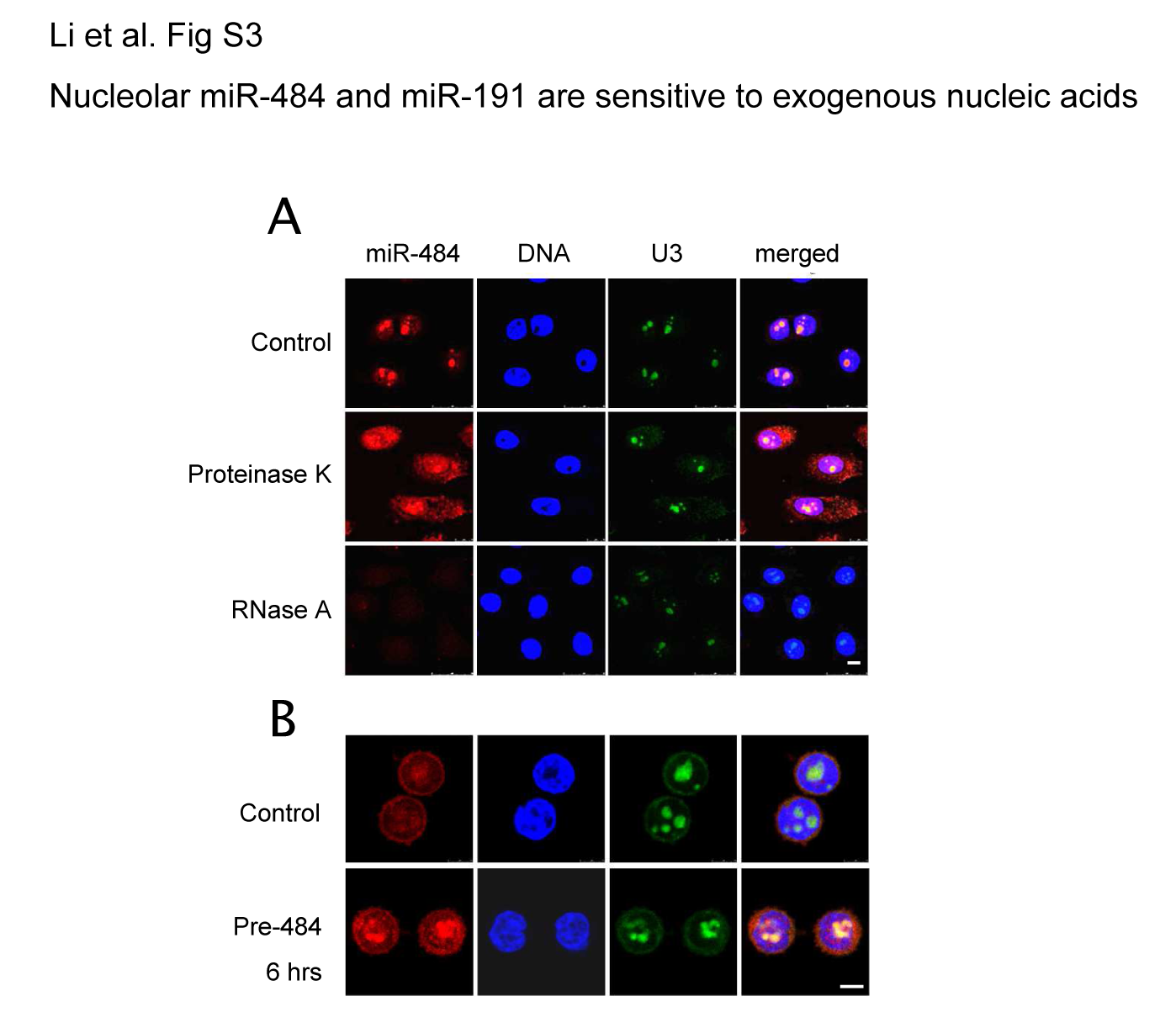

Supplement: Figure S3 — ISH signal of miR-484 is sensitive to RNase A and delivery of pre-miR-484 increases nucleolar miR-484 in HeLa cells. Cells are incubated for 20 min at room temperature with either PBS (mock treatment), RNase A (100 µg/ml, Invitrogen) or Proteinase K (100 µg/ml, Promega) and further fixed for ISH staining. The HeLa cells are also transfected with 25 nM pre-miR-484 by a NEON transfection system. Post transfection (6 hrs), they are fixed and analysed by ISH staining. Nucleolar marker, U3 is in green and cell nuclei are stained with HOECHST33258. Scale bars, 10 µm. (TIF) [file pone.0070869.s003.tif]

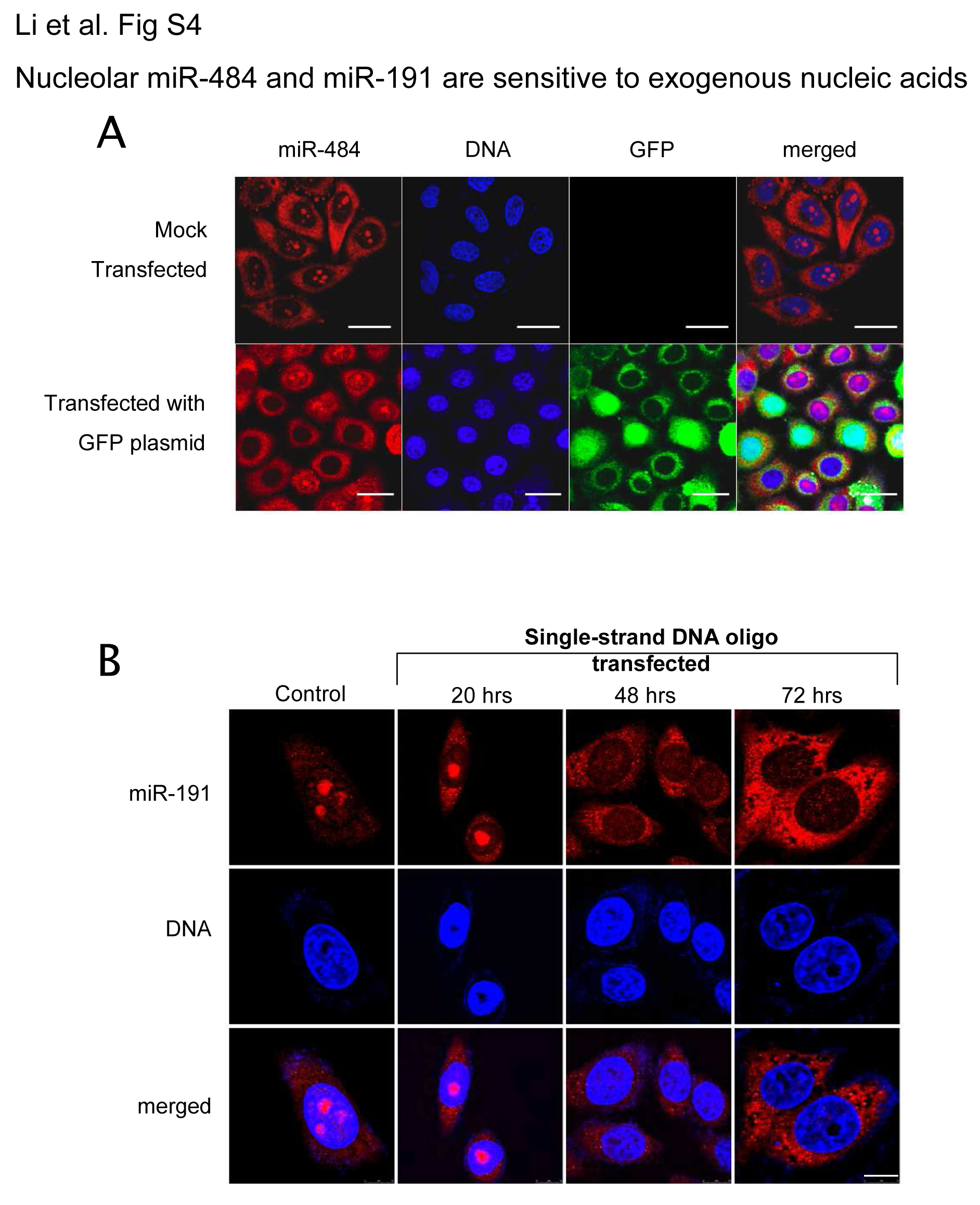

Supplement: Figure S4 — Nucleolars miR-484 and miR-191 are sensitive to exogenous nucleic acids. The HeLa cells were mock-transfected (A, upper panels) or transfected with 1 µg/ml pCDNA-GFP plasmid (A, lower panels) for 72 hrs, or transfected with a single stranded short DNA oligo for 20 hrs, 48 hrs and 72 hrs (B), and fixed and processed for ISH with an miR-484 or miR-191 probe. The expression of the GFP is also captured (A). The cell nuclei are stained with HOECHST33258. Scale bars: A, 25 µm, B, 10 µm. (TIF) [file pone.0070869.s004.tif]

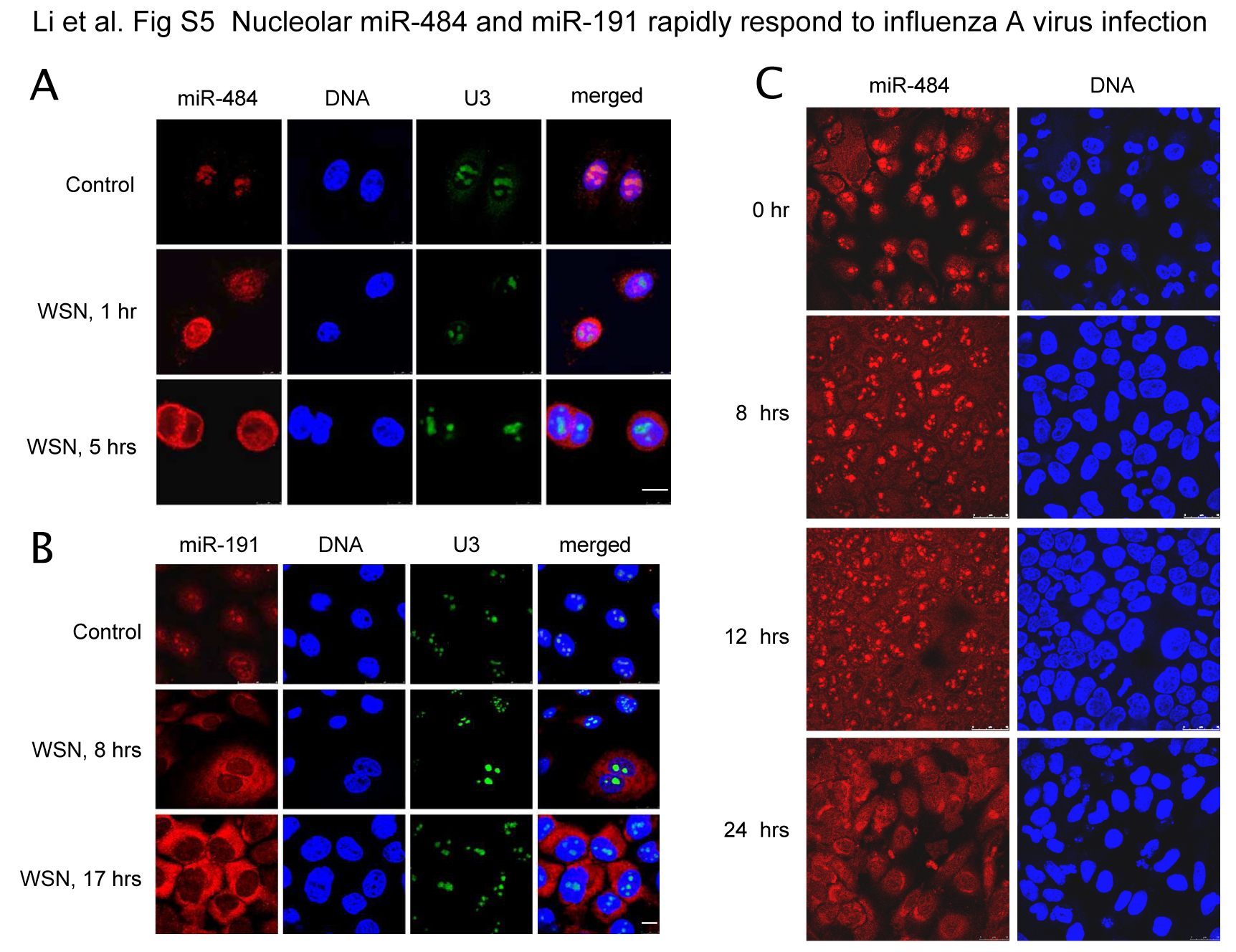

Supplement: Figure S5 — Nucleolars miR-484 and miR-191 rapidly respond to influenza A virus infection. Influenza A model cell lines A549 - a human lung cancer cell (A) and MDCK- a dog kidney cells (B, C) are infected with influenza A/WSN for different times, fixed and processed for ISH analysis with miR-191 and miR-484 probes. The cell nucleoli are indicated with U3 snoRNA (green) and the cell nuclei are stained with HOECHST33258 (Blue). Scale bars, 10 µm. (TIF) [file pone.0070869.s005.tif]
