# Supplementary material for: Dynamic Localisation of Mature MicroRNAs in Human Nucleoli is Influenced by Exogenous Genetic Materials
Source: PLoS One. 2013 Aug 6;8(8):e70869. doi: 10.1371/journal.pone.0070869 (PMC3735495; doi:10.1371/journal.pone.0070869)
Supplement: Table S2 — Nucleolar miRNAs in HeLa. The genomic location, validated targets and sequence of the most abundant 11 nucleolar miRNAs were summarized. (DOC) [file pone.0070869.s007.doc]

**Table S2. Nucleolar miRNAs in HeLa**

| **miRNA** | **Genomic**  **location** | **Previously**  **Identified** | **Validated Targets** | **Sequence** |
| --- | --- | --- | --- | --- |
| **miR-191** | 3p21 | Nucleolus | RIOK3, FANCD2, MXI1, MLH1, et al. | 5’-CAACGGAAUCCCAAAAGCAGCUG-3’ |
| **miR-484** | 16p13 | Nucleolus | POMC, MATR3, HSF1 | 5’-UCAGGCUCAGUCCCCUCCCGAU-3’ |
| **miR-193b** | 16p |  | SOX9, MYC, BCL2, MCL1 et al. | 5’-AACUGGCCCUCAAAGUCCCGCU-3’ |
| **miR-574-3p** | 4p14 |  | DICER1, CDKN2A, AFP, IFG2R et al. | 5’-CACGCUCAUGCACACACCCACA-3’ |
| **miR-454** | 17q22 |  | SLAIN1 | 5’-ACCCUAUCAAUAUUGUCUCUGC-3’ |
| **miR-125a-5p** | 19q13.41 | Nucleolus | LFNG, DICER1, CD83, BRCA1 et al. | 5’-UCCCUGAGACCCUUUAACCUGUGA-3’ |
| **miR-342-3p** | 14q32.2 |  | IL7R, CD4, BCL2, EGFR et al. | 5’-AGGGGUGCUAUCUGUGAUUGA-3’ |
| **miR-1275** | 6p33.9 |  | CLDN11 | 5’-GUGGGGGAGAGGCUGUC-3’ |
| **miR-1260** | 14 |  | NA | 5’-AUCCCACCUCUGCCACCA-3’ |
| **miR-196b** | 7p15.2 |  | HOXA10, ANXA1, BMP4, CD33 et al. | 5’-UAGGUAGUUUCCUGUUGUUGGG-3’ |
| **miR-93#** | 7q22.1 |  | STAT3, PAK3, ZNFX1, TBK1 et al. | 5’-CAAAGUGCUGUUCGUGCAGGUAG-3’ |
| **miR-1274a** | 5 | Nucleolus | NA | tRNA degrade |
| **miR-1274b** | 19q13.43 | Nucleolus | NA | tRNA degrade |
